# Influence of Cardiovascular Risk Factors, Comorbidities, Medication Use and Procedural Variables on Remote Ischemic Conditioning Efficacy in Patients with ST-Segment Elevation Myocardial Infarction

**DOI:** 10.3390/ijms20133246

**Published:** 2019-07-02

**Authors:** Kasper Pryds, Marie Vognstoft Hjortbak, Michael Rahbek Schmidt

**Affiliations:** 1 Department of Cardiology, Aarhus University Hospital, 8200 Aarhus N, Denmark; 2 Department of Clinical Medicine, Aarhus University, 8200 Aarhus N, Denmark; 3 Department of Medicine, Randers Regional Hospital, 8930 Randers, Denmark; 4 Department of Cardiology, Rigshospitalet, 2100 Copenhagen Ø, Denmark

**Keywords:** acute myocardial infarction, ST-segment elevation myocardial infarction, post-infarction heart failure, remote ischemic conditioning, ischemic preconditioning

## Abstract

Remote ischemic conditioning (RIC) confers cardioprotection in patients with ST-segment elevation myocardial infarction (STEMI). Despite intense research, the translation of RIC into clinical practice remains a challenge. This may, at least partly, be due to confounding factors that may modify the efficacy of RIC. The present review focuses on cardiovascular risk factors, comorbidities, medication use and procedural variables which may modify the efficacy of RIC in patients with STEMI. Findings of such efficacy modifiers are based on subgroup and post-hoc analyses and thus hold risk of type I and II errors. Although findings from studies evaluating influencing factors are often ambiguous, some but not all studies suggest that smoking, non-statin use, infarct location, area-at-risk of infarction, pre-procedural Thrombolysis in Myocardial Infarction (TIMI) flow, ischemia duration and coronary collateral blood flow to the infarct-related artery may influence on the cardioprotective efficacy of RIC. Results from the on-going CONDI2/ERIC-PPCI trial will determine any clinical implications of RIC in the treatment of patients with STEMI and predefined subgroup analyses will give further insight into influencing factors on the efficacy of RIC.

## 1. Introduction

Even though the relative incidence of ST-segment elevation myocardial infarction (STEMI) is decreasing [1], the mortality is high with in-hospital mortality varying from 4% to 12% across European countries [2]. Among Danish patients with STEMI treated with primary percutaneous coronary intervention (PPCI), 30-day, 1-year and 5-year cardiac mortality rates have been found to be 7%, 8% and 14%, respectively [3]. Despite major improvement in acute treatment of patients with acute myocardial infarction (AMI) during the last decades leading to significant improvement in mortality [4], hospitalization for post-infarction heart failure has not decreased as expected [5]. Thus, challenges remain to improve the clinical outcome after STEMI. 

STEMI results from abrupt total coronary artery occlusion causing myocardial ischemia and is most often caused by coronary atherosclerotic plaque rupture precipitating intracoronary thrombus formation. Ischemia duration is a main determinant of the extent of myocardial cellular death and infarct size [6]. Consequently, timely and sufficient reperfusion of the coronary artery by PPCI or thrombolysis is essential in order to salvage ischemic myocardium in patients with STEMI [7]. However, reperfusion itself may cause excessive de novo myocardial cellular damage [8]. This phenomenon is referred to as ischemia-reperfusion (I-R) injury and is a key component in STEMI. In addition to myocardial cellular death, I-R injury may also cause endothelial and vasomotor dysfunction, deterioration of contractile function and malignant arrhythmias [9,10]. While continued optimisation of patient referral logistics remains the primary strategy to reduce ischemia duration, it does not abrogate reperfusion injury. Moreover, notwithstanding improvement in healthcare logistics, extended treatment delay remains a challenge in a subset of patients. Consequently, there is a need for adjunctive treatment strategies targeting the detrimental effects of I-R injury in order to further improve the outcome for patients with STEMI [11].

Myocardial sensitivity to ischemia is modifiable by both pharmacological and non-pharmacological interventions, which may substantially reduce myocardial cellular death. Such strategies are referred to as *cardioprotection*. A vast number of adjunctive treatment strategies have been found to confer cardioprotective effect in experimental and proof of concept clinical studies in patients with STEMI [12,13]. One of the most promising cardioprotective strategy to achieve protection against I-R injury is remote ischemic conditioning (RIC). RIC is achieved by brief intermittent cycles of ischemia and reperfusion of a distant organ prior, during or following myocardial I-R [14]. Despite intense research, the translation of RIC into clinical practice remains a challenge [11,15]. A number of confounding factors may modify the efficacy of RIC in patients with STEMI [16,17,18]. For successful clinical translation of RIC, it is important to identify any factor influencing the efficacy of RIC [19]. Thus, the present review focuses on cardiovascular risk factors, comorbidities, medication use and PPCI-procedural variables, which may modify the efficacy of RIC in patients with STEMI. 

To identify clinical studies investigating the effect of RIC in patients with STEMI, we performed a systematic search of the PubMed database using the MeSH terms “STEMI,” “thrombolysis” and “primary percutaneous intervention,” in combination with either “remote ischemic conditioning,” “remote ischemic preconditioning,” “remote ischemic preconditioning” or “remote ischemic postconditioning.” Furthermore, reviews on the topic were studied for relevant references [15,20,21,22]. Eligibility criteria were original studies investigation the clinical effect of RIC in a setting of STEMI. English language studies with available full text were reviewed, of which 16 were found relevant and included in the present review (Table 1).

## 2. The Concept of RIC

A detailed description of the molecular and clinical aspects of RIC is beyond the scope of the present review. For detailed description of the concept of RIC and its clinical implications, readers may want to consult excellent reviews by Heusch and colleagues [14,41]. Briefly, in 1986, Murry and colleagues first demonstrated that protection against ischemic myocardial injury could be achieved by application of transient episodes of brief, sublethal episodes of myocardial I-R prior to an episode of lethal I-R injury [42]. In 1993, Przyklenk and colleagues introduced the concept that we now know as RIC by demonstrating that the application of transient episodes of brief, sublethal episodes of myocardial I-R also protects remote myocardial territories against lethal I-R injury [43]. Later, Kharbanda and colleagues translated protective effects from RIC to humans by application of transient episodes of I-R to the upper limb [44]. In an experimental porcine model of myocardial infarction, Schmidt and colleagues found that RIC was cardioprotective even if applied during evolving myocardial ischemia [45], rendering RIC a clinical relevant adjunctive treatment strategy in situations of unpredictable ischemia, such as STEMI [11]. 

The underlying signalling pathways and molecular mechanisms of RIC are still not fully understood. However, the cardioprotective effect by RIC involves both humoral and neuronal signalling pathways and activation of protective intracellular signalling pathways in the target organ [14]. A number of protective signalling pathways and mediators have been found to be involved in the signal transduction of RIC [41]. The reperfusion injury salvage kinase pathway seems of particular importance for the cardioprotective effects of RIC [14] through its protective effects on the mitochondria at time of myocardial reperfusion. This, at least partly, by inhibiting opening of the mitochondrial permeability transition pore [46], which appears a final common pathway determinant of cellular death by I-R injury [11]. 

## 3. Effect of RIC in Patients with STEMI

In the clinical setting, application of RIC by short-term intermittent episodes of I-R is most often applied on the upper-arm and induced by alternating inflations and deflations of a standard blood pressure cuff above systolic blood pressure. In patients with STEMI treated with PPCI, proof of concept studies have demonstrated that RIC reduces cardiac biomarker release [27,28,38] and infarct size [30], improves myocardial salvage [24,31], that is, the proportion of the ischemic myocardium at risk of infarction being salvaged by the assigned treatment [47], ST-segment resolution on electrocardiogram [23] and peripheral endothelial function [29], as well as reduce the incidence of acute kidney injury [39]. Most importantly RIC reduces cardiac mortality and rehospitalisation for heart failure [40]. RIC has also been found to reduce cardiac biomarker release [32] and increase ST-segment resolution [36] in patients treated with thrombolysis (Table 1). A meta-analysis based on available clinical proof of concept studies indicates that RIC confers cardioprotective effect in patients with STEMI in terms of surrogate endpoints, that is, reduction in cardiac biomarker release and ST-segment resolution, as well as all-cause mortality [48]. Moreover, post hoc exploratory studies indicate that RIC may be cost-effective [49] and improve long-term clinical outcome [50] in patients with STEMI treated with PPCI. Importantly, findings on the effects of RIC on the included secondary study endpoints are ambiguous and none of the studies conducted to date have been sufficiently powered to evaluate the effect of RIC on clinical outcomes [11]. Consequently, the on-going large-scale multicentre trial CONDI2/ERIC-PPCI (clinicaltrials.gov Identifiers: NCT01857414 and NCT02342522) is important to clarify clinical implications of RIC as an adjunctive to PPCI in patients with STEMI [51]. In the meantime, subgroup and post-hoc analyses have revealed patient characteristics and PPCI-procedural variables, which may modify the efficacy of RIC in patients with STEMI and therefore should be considered in any attempt to translate the beneficial effect of RIC into the clinical setting. 

In addition to conferring beneficial effect when applied at the time of the STEMI in relation to reperfusion treatment, an experimental study suggests that repeated RIC application applied immediately following an AMI may yield additional beneficial anti-remodelling effect and improve survival [52]. This promising clinical implication of RIC treatment is currently being investigating in the clinical setting (clinicaltrials.gov Identifier: NCT01817114). Moreover, we have found that repeated RIC application may also have beneficial effects on myocardial and skeletal muscle function [53], as well as mild anti-inflammatory, cardiac remodelling [54] and fibrinolytic effects [53] in patients with stable chronic ischemic heart failure. 

## 4. Influence of Cardiovascular Risk Factors, Patient Comorbidities and Characteristics and Medication Use on the Efficacy of RIC

In experimental settings, a number of cardiovascular risk factors, comorbidities and concomitant pharmacological treatment have been found to influence on I-R severity, for example, aging, diabetes and hyperlipidaemia [16] and may also influence the efficacy of RIC in patients with STEMI. In an experimental and proof of concept clinical study on healthy volunteers, we have demonstrated that the efficacy of RIC on I-R injury is attenuated by long-term treatment with glyceryl trinitrate [55].

The influence of cardiovascular risk factors, patient comorbidities and concomitant medication use on clinical RIC efficacy on the primary study endpoint has been investigated in studies by Crimi and colleagues (age, sex, diabetes mellitus and multivessel disease) [27], Eitel and colleagues (age, sex and Killip class at time of presentation) [31], Verouhis and colleagues (smoking) [33], Ladejobi and colleagues (cardiac arrest at time of presentation) [35] and in a post-hoc analysis of the CONDI trial by Sloth and colleagues (age, sex, smoking, body mass index, diabetes mellitus, hypertension, left ventricular hypertrophy, blood cholesterol and glucose levels and use of beta-blockers, angiotensin converting enzyme inhibitors, angiotensin II receptor blockers, calcium channel blockers and statins) [56] (Table 1). Of particular importance, high Killip class [57] as well as complicating cardiac arrest [58] are associated with worse clinical outcome in patients with STEMI. In this light, intriguing findings on the effect of RIC have been demonstrated by Eitel and Ladejobi and colleagues, as neither Killip class [31] nor cardiac arrest at the time of presentation [35] were found to influence on the efficacy of RIC on myocardial salvage or incidence of in-hospital heart failure, respectively. No other significant effect modification from cardiovascular risk factors, patient comorbidities or medication use was found in any of the studies. However, there was a trend towards negative effect medication from smoking and in non-statin users on the efficacy of RIC on myocardial salvage in a post-hoc analysis by Sloth and colleagues based on the CONDI trial [56]. Notably, in the RIC-STEMI trial by Gaspar and colleagues, RIC improved the secondary endpoint of left ventricular ejection fraction (LVEF) by an absolute of 10% compared to PPCI alone in the subset of patients presenting with reduced LVEF at the time of STEMI [40]. 

### Pre-Infarction Angina

Pre-infarction angina (PIA) describes the occurrence of symptomatic angina pectoris episodes preceding the onset of an AMI and represents brief I-R episodes of the myocardium similar to that of ischemic preconditioning [59]. Potentially, PIA could confer cardioprotective effect *per se* and in this way influence on the cardioprotective efficacy of RIC in patients with STEMI. Incidence of PIA within 48 hours prior to the onset of AMI occur in 3% to 40% of patients [60,61,62,63]. In patients with STEMI referred to PPCI, PIA may reduce cardiac biomarker release [61,64,65] and increase myocardial salvage [66], whereas effects on infarct size [66,67] and mortality [61,62,64] are ambiguous. Of note, studies suggest that PIA may confer the most pronounced cardioprotective effect when closely preceding the AMI [63,68]. Consequently, the timing of PIA in relation to the onset of AMI [68,69,70] as well as the choice of reperfusion strategy [59] may influence on the cardioprotective effect of PIA [11]. 

In a post-hoc analysis of the CONDI trial, we found no cardioprotective of PIA *per se* on myocardial salvage in patients with STEMI treated with PPCI [26]. More importantly, PIA did not influence on the cardioprotective efficacy of RIC. This is compatible with findings on the efficacy of RIC on cardiac biomarker release in patients with anterior STEMI in the study by Crimi and colleagues [27]. 

In conclusion, data regarding the influence of cardiovascular risk factors, patient comorbidities and medication use on the efficacy of RIC are limited and rely mainly on animal studies and insufficiently powered post-hoc analyses. Thus, findings should be interpreted with caution. However, available data suggest that smoking and non-statin use might influence negatively on the efficacy of RIC. Moreover, there seems to be a beneficial effect of RIC on LVEF in the subset of patients presenting with reduced LVEF at the time of STEMI. 

## 5. Influence of PPCI-Procedural Variables on the Efficacy of RIC

### 5.1. Infarct Location, AAR of Infarction, Pre-Procedural TIMI Flow and Other PPCI-Procedural Variables

In patients with STEMI, infarct location and pre-procedural Thrombolysis In Myocardial Infarction (TIMI) flow are of significant importance because patients presenting with left anterior descending artery-infarction or low pre-procedural TIMI flow are at increased risk of post-infarction heart failure evolvement [71]. In the CONDI trial, area-at-risk (AAR) of infarction as well as the primary endpoint of myocardial salvage were assessed by single-photon emission computed tomography (SPECT) as a percentage of the left ventricle and left anterior descending artery-infarction was associated with more extensive AAR of infarction [24]. In that study, the cardioprotective effect of RIC was preserved for patients presenting with left anterior descending artery-infarction. Moreover, the efficacy of RIC was found to correlate with AAR, that is, the greater AAR the more pronounced effect of RIC on myocardial salvage. These findings are supported by the RIC-STEMI trial conducted by Gaspar and colleagues in which the efficacy of RIC on the combined endpoint of cardiac mortality or rehospitalization for heart failure was most pronounced among patients with anterior myocardial infarction. These observations are likely explained by the notion that the larger myocardial AAR of infarction, the greater the potential for cardioprotection by RIC. However, the studies by Eitel and colleagues and Verouhis and colleagues found no effect modification from AAR of infarction [31] or infarct location [31,33] on myocardial salvage assessed by cardiac magnetic resonance (CMR) imaging. However, inherent differences in AAR quantification by SPECT and CMR imaging may compromise comparison of findings on MSI from the CONDI trial and studies by Eitel and Verouhis and colleagues. Irrespective of findings relying on myocardial salvage, Munk and colleagues conducted a substudy of the CONDI trial evaluating the effect of RIC on myocardial contractile function in terms of LVEF assessed by echocardiography [72]. Compatible with the findings on myocardial salvage in the parent trial, the authors demonstrated that RIC improved LVEF by an absolute 5% but that this effect was preserved the subset of patients with extensive AAR of infarction or patients presenting with anterior myocardial infarction. 

In the CONDI trial, the cardioprotective effect of RIC on myocardial salvage was predominantly observed among patients with pre-procedural TIMI flow grade 0-1 [24] and among patients with pre-procedural TIMI flow grade 0 in the RIC-STEMI trial by Gaspar and colleagues [40]. In contrast, neither Eitel and colleagues nor Verouhis and colleagues observed effect modification from pre-procedural TIMI flow [31,33]. These findings suggest that comprised blood flow of the infarct-related artery may not be a prerequisite for a cardioprotective effect of RIC. On the other hand, intuitively, any cardioprotective potential of RIC would be diminished in the case of very early spontaneous restoration of coronary blood flow prior to reperfusion with PPCI or thrombolysis, or if the spontaneous restoration of coronary blood flow precedes the application of RIC. 

Differences in applied PPCI techniques, that is, use of aspiration thrombectomy or direct stenting, do not seem to influence on the efficacy of RIC on myocardial salvage based on findings in the study by Eitel and colleagues [31]. However, no other study has evaluated effect modification from variations in PPCI techniques. Consequently, these findings should be interpreted with caution.

### 5.2. Coronary Collateral Blood Flow to the Infarct-Related Artery

The coronary arteries are connected by a network of coronary collateral vessels [73]. The coronary collateral circulation serves as conduits to coronary territories with partial or complete obstruction of the originally supplying artery. A coronary collateral circulation sufficient to prevent myocardial ischemia has been found to be present in 33% and 20–25% of individuals with and without ischemic heart disease, respectively [74]. In patients with STEMI treated with PPCI, coronary collateral blood flow (CCBF) to the infarct-related artery seems to reduce cardiac biomarker release [19,75,76,77,78,79,80], evolving cardiogenic shock [79] and post-infarction heart failure [77]. In contrast, the cardioprotective effect from CCBF remains controversial with regards to infarct size [66,76,78,81,82], myocardial salvage [66,78], myocardial contractile function [66,76,77,78,80,82,83] and mortality [75,76,77,79,80,84]. Repetitive episodes of myocardial ischemia, that is, ischemic preconditioning, induce an immediate increase in CCBF to the occluded artery [85,86], which has been found to correlate with the degree of cellular tolerance to ischemia [85]. However, only a minority of ischemic tolerance seems related to coronary collateral recruitment, whereas the majority would be the result of ischemic preconditioning [42,85]. No study has evaluated whether RIC per se modulates the coronary collateral circulation. Notably, in our post-hoc analysis of the CONDI trial, no association was observed between RIC treatment and the prevalence of CCBF to the infarct-related artery [26], suggesting that RIC does not confer cardioprotection through coronary collateral recruitment [11].

In our post-hoc analysis of the CONDI trial, we found that the efficacy of RIC on myocardial salvage may correlate with the degree of CCBF to the infarct-related artery [26]. Our findings support the notion that circulating cardioprotective humoral factors are centrally involved in cardioprotection by RIC [14]. We used SPECT for assessment of AAR of infarction and myocardial salvage. However, because we defined AAR of infarction using a cut-off determined by myocardial perfusion tracer uptake we were not able to correlate myocardial ischemia severity or the functional capacity of CCBF to the infarct-related artery to the efficacy of RIC. Modifiable vasomotor capacity of the coronary collateral circulation [87] might contribute to protective effects by RIC. Studies by Prunier and colleagues [28], Crimi and colleagues [27] and White and colleagues [30] found cardioprotective effect of RIC on cardiac biomarker release and infarct size using CMR among patients with no or little CCBF to the infarct-related artery. Importantly, these findings suggest that CCBF to the infarct-related artery may not be a prerequisite for achieving cardioprotection by RIC [11]. 

### 5.3. Medication Administration in Relation to PPCI and PPCI Technique

As with RIC, early experimental data suggests that opioid receptor activation confer cardioprotection by inhibiting mitochondrial permeability transition pore opening [88]. Rentoukas and colleagues investigated the cardioprotective effect of RIC and a combination of RIC with pre-PPCI morphine administration on ST-segment resolution on electrocardiogram in patients with STEMI [23]. While RIC itself was found to confer cardioprotective effect, there was a trend, although not statistically significant, towards positive effect modification by morphine administration at the time of RIC application. However, this finding was not confirmed by Crimi and colleagues on cardiac biomarker release [27]. 

Eitel and colleagues found no effect modification from glycoprotein IIb/IIIa inhibitor administration at the time of PPCI on the efficacy of RIC on myocardial salvage [31].

### 5.4. Ischemia Duration and Health-Care System Delay

Treatment delay for patients with STEMI is defined as the time from symptom onset to reperfusion therapy, that is, PPCI or thrombolysis and reflects total myocardial ischemia duration [89]. Treatment delay reflects both patient delay, that is, the time from symptom onset to first medical contact and healthcare system delay, that is, the time from first medical contact to reperfusion therapy [90]. While treatment delay holds risk of recall bias and therefore is difficult to determinate accurately, healthcare system delay assessment is objective and less variable for comparison use [90]. Compared to treatment delay, healthcare system delay may better predict adverse clinical outcome [91]. Consequently, healthcare system delay is increasingly being used as surrogate for ischemia duration in patients with STEMI. Healthcare system delay is a negative independent predictor of left ventricle contractile function [92], myocardial salvage [25,92], infarct size [92] and clinical outcome in terms of return to labour market and work retirement [93], rehospitalisation for heart failure [94] and mortality [91,95]. Despite up-to-date prehospital diagnostics and field-triage, extensive healthcare system delays remain a challenge for a significant number of patients with STEMI. This is mainly a result of long distances to PPCI-centres. In addition to having detrimental effects in itself, the duration of on-going myocardial ischemia may also critically influence the efficacy of RIC. Of significant importance, in a post-hoc analysis of the CONDI trial, we found that prehospital application of RIC by ambulance personnel did not prolong healthcare system delay for patients with STEMI referred to PPCI [25]. Moreover, we found effect modification from healthcare system delay on the efficacy of RIC, implying that the efficacy of RIC on myocardial salvage increased with the duration of healthcare system delay [25]. Our findings suggest that the cardioprotective efficacy of RIC may be critically dependent on the duration of on-going myocardial ischemia [11]. This notion is supported by findings in the RIC-STEMI trial by Gaspar and colleagues [40] and the study by Eitel and colleagues [31]. In the RIC-STEMI trial, the efficacy of RIC on the combined endpoint of cardiac mortality or rehospitalization for heart failure was most pronounced among patients with treatment delay >3 hours. Similar findings, although not statistically significant, were observed by Eitel and colleagues on the cardioprotective effect of RIC in combination with local ischemic postconditioning on myocardial salvage [31]. In contrast, Crimi and colleagues [27] and Verouhis and colleagues [33] found no effect modification from ischemia duration on enzymatic infarct size and myocardial salvage, respectively. Despite these ambiguous results, it is possible that a certain ischemic threshold needs to be reached in order for RIC to be effective. On the other hand, the effect of RIC could be compromised by very extended ischemic duration because of loss of reversibility and hence any potential for cardioprotection when irreversibility ensues [11]. In this light, it is likely that ischemia duration in its most extreme entities indeed does influence on cardioprotective efficacy of RIC.

In conclusion, studies evaluating influence of PPCI-procedural variables on the efficacy of RIC in patients with STEMI are ambiguous. Some but not all studies suggest that RIC is most effective in patients with left anterior descending artery-infarction, extensive AAR of infarction or ischemia duration or with an occluded infarct-related coronary artery at the time of PPCI. Moreover, the efficacy of RIC may correlate with the degree of CCBF to the infarct-related artery.

## 6. Conclusions

Findings on influencing factors on the efficacy of RIC in patients with STEMI depend on subgroup and post-hoc analyses, hold risk of type I and II errors and yield ambiguous results. However, some studies suggest that smoking, non-statin use, infarct location, AAR of infarction, pre-procedural TIMI flow, ischemia duration and CCBF to the infarct-related artery may influence on the cardioprotective efficacy of RIC. Results from the on-going CONDI2/ERIC-PPCI trial will clarify clinical implications of RIC in the treatment of patients with STEMI and predefined subgroup analyses will give further insight into confounding factors on its efficacy. Beyond the beneficial effect as an adjunct to revascularization in patients with STEMI, experimental and clinical pilot studies suggest that repeated RIC application may yield further beneficial effect in the post-infarction period and in patients with chronic ischemic heart failure.

## Figures and Tables

**Table 1:** Overview of Clinical Studies on the Effect of remote ischemic conditioning (RIC) and Influencing Factors on Primary Cardiovascular Endpoints in Patients with ST elevation myocardial infarction (STEMI).

Study	Patients	RIC Protocol	Effect of RIC on Primary Endpoint	Variables Influencing Efficacy of RIC on Primary Endpoint
Rentoukas [23], 2010	*n* = 96	3 cycles of 4 min I-R of the upper arm during PPCI	Increased ST-segment resolution on electrocardiogram	N/A
Bøtker [24], 2010	*n* = 333	4 cycles of 5 min I-R of the upper arm prior to PPCI	Increased MSI assessed by SPECT	Effect modification from LAD artery infarction, AAR, pre-procedural TIMI flow, ischemia duration [25] and CCBF [26]. No observed effect modification from PIA [26], cardiovascular risk factors or concomitant medication use
Crimi [27], 2013	*n* = 100, only patients with LAD artery infarction, pre-procedural TIMI flow grade 0-1 and without CCBF	3 cycles of 5 min I-R of the lower limb following PPCI	Reduction in cardiac biomarker release assessed by CK-MB	No observed effect modification from age, sex, diabetes mellitus, PIA, morphine administration, ischemia duration or multivessel disease
Prunier [28], 2014	*n* = 151, only patients with LAD or RCA artery infarction, pre-procedural TIMI flow grade 0-1 and without significant CCBF	3 cycles of 5 min I-R of the upper arm prior to PPCI	Reduction in cardiac biomarker release assessed by CK-MB	N/A
Manchurov [29], 2014	*n* = 48, both patients with STEMI and NSTEMI	4 cycles of 5 min I-R of the upper arm prior to PPCI	Improvement in peripheral endothelial function assessed by brachial artery flow-mediated dilation	N/A
White [30], 2015	*n* = 197, only patients with pre-procedural TIMI flow grade 0 and without CCBF	4 cycles of 5 min I-R of the upper arm prior to PPCI	Reduction in MI size assessed by CMR	N/A
Eitel [31], 2015	*n* = 696	3 cycles of 5 min I-R of the upper arm prior to PPCI + ischemic postconditioning	Reduction in MSI assessed by CMR	No observed effect modification from sex, age, infarct location, AAR, pre-procedural TIMI flow, ischemia duration, Killip class, thrombectomy, direct stenting or GP IIb/IIIa inhibitor administration
Yellon [32], 2015	*n* = 519	4 cycles of 5 min I-R of the upper arm prior to thrombolysis	Reduction in cardiac biomarker release assessed by troponin T and CK-MB	N/A
Verouhis [33], 2016	*n* = 150, only patients with anterior infarction and without concomitant treatment with glibenclamide or cyclosporine	Cycles of 5 min I-R of the lower limb prior to and during PPCI	No effect on MSI assessed by CMR	No observed effect modification from smoking, LAD artery infarction, pre-procedural TIMI flow or ischemia duration
Lotfollahi [34], 2016	*n* = 82, only patients with age <80 years	3 cycles of 4 min I-R of the upper arm during PPCI	Reduction in serum oxidative stress assessed by glutathione peroxidase	N/A
Ladejobi [35], 2017	*n* = 150, only patients with LVEF <55% and without hypotension	4 cycles of 5 min I-R of the upper arm prior to PPCI	Reduction in incidence of in-hospital HF	No observed effect modification from presenting with cardiac arrest
Ghaffari [36], 2017	*n* = 78	3 cycles of 5 min I-R of the upper arm during to thrombolysis	Increased ST-segment resolution on electrocardiogram	N/A
Elbadawi [37], 2017	*n* = 71, only patients with anterior infarction	3 cycles of 5 min I-R of the lower limb following PPCI	No effect on adverse left ventricular remodelling or LVEF assessed by echocardiography	N/A
Cao [38], 2018	*n* = 80, only patients without concomitant treatment with trimethoprim or glibenclamide	4 cycles of 5 min I-R of the upper arm following PPCI	Reduction in cardiac biomarker release assessed by CK-MB	N/A
Cao [39], 2018	*n* = 72, only patients without concomitant treatment with trimethoprim or glibenclamide	4 cycles of 5 min I-R of the upper arm following PPCI	Reduction in acute kidney injury assessed by serum creatinine	N/A
Gaspar [40], 2018	*n* = 258	3 cycles of 5 min I-R of the lower limb during PPCI	Reduction in the combined endpoint of cardiac mortality or rehospitalization for HF at 2-year follow-up	Effect modification from anterior MI, pre-procedural TIMI flow and ischemia duration

RIC, Remote ischemic conditioning; I-R, Ischemia-reperfusion; PPCI, Primary percutaneous coronary intervention; MSI, Myocardial salvage index; SPECT, Single-photon emission computed tomography; LAD, Left anterior descending; AAR, Area-at-risk; TIMI, Thrombolysis in myocardial infarction; RCA, Right coronary artery; CCBF, Coronary collateral blood flow; PIA, Pre-infarction angina; NSTEMI, Non-ST-segment elevation myocardial infarction; MI, Myocardial infarct; CK-MB, Creatine kinase-myocardial band; CMR, Cardiac magnetic resonance; LVEF, Left ventricular ejection fraction; HF, Heart failure; MI, Myocardial infarction; GP, Glycoprotein; N/A, Not applicable.

## Abbreviations

AAR	Area-at-risk
AMI	Acute myocardial infarction
CCBF	Coronary collateral blood flow
CMR	Cardiac magnetic resonance
I-R	Ischemia-reperfusion
LVEF	Left ventricular ejection fraction
PIA	Pre-infarction angina
PPCI	Primary percutaneous coronary intervention
RIC	Remote ischemic conditioning
SPECT	Single-photon emission computed tomography
STEMI	ST-segment elevation myocardial infarction
TIMI	Thrombolysis In Myocardial Infarction
